# Rapid, Sensitive, and Species-Specific Detection of Conventional and Recombinant Herpesvirus of Turkeys Vaccines Using Loop-Mediated Isothermal Amplification Coupled With a Lateral Flow Device Readout

**DOI:** 10.3389/fvets.2022.873163

**Published:** 2022-06-23

**Authors:** Giulia Mescolini, Susan J. Baigent, Elena Catelli, Venugopal K. Nair

**Affiliations:** ^1^Avian Pathology Service, Department of Veterinary Medical Sciences, University of Bologna, Bologna, Italy; ^2^Viral Oncogenesis Group, The Pirbright Institute, Woking, United Kingdom

**Keywords:** Marek's disease, herpesvirus of turkeys, LAMP-LFD, FC126, Vaxxitek®

## Abstract

Marek's disease, an economically important disease of chickens caused by virulent serotype 1 strains of the *Mardivirus* Marek's disease virus (MDV-1), is effectively controlled in the field by live attenuated vaccine viruses including herpesvirus of turkeys (HVT)—both conventional HVT (strain FC126) and, in recent years, recombinant HVT viruses carrying foreign genes from other avian viruses to protect against both Marek's disease and other avian viral diseases. Testing to monitor and confirm successful vaccination is important, but any such test must differentiate HVT from MDV-1 and MDV-2, as vaccination does not prevent infection with these serotypes. End-point and real-time PCR tests are widely used to detect and differentiate HVT, MDV-1 and MDV-2 but require expensive specialist laboratory equipment and trained operators. Here, we developed and validated two tube-based loop-mediated isothermal amplification tests coupled with detection by lateral flow device readout (LAMP-LFD): an HVT-specific test to detect both conventional and recombinant HVT strains, and a second test using novel LAMP primers to specifically detect the Vaxxitek® recombinant HVT. Specificity was confirmed using DNA extracted from virus-infected cultured cells, and limit of detection was determined using plasmid DNA carrying either the HVT or Vaxxitek® genome. The LAMP-LFD tests accurately detected all HVT vaccines, or Vaxxitek® only, in crude DNA as well as purified DNA extracted from field samples of organs, feathers, or poultry house dust that were confirmed positive for HVT by real-time PCR. These LAMP-LFD tests have potential for specific, rapid, simple, and inexpensive detection of HVT vaccines in the field.

## Introduction

*Meleagrid alphaherpesvirus 1*, the herpesvirus of turkeys (HVT), is a member of the genus *Mardivirus* of the *Alphaherpesvirinae* subfamily (1), together with *Gallid alphaherpesvirus 2*, traditionally referred to as Marek's disease virus serotype 1 (MDV-1), the aetiological agent of Marek's disease (MD), and *Gallid alphaherpesvirus 3*, or Marek's disease virus serotype 2 (MDV-2). HVT was isolated for the first time in 1968 from healthy turkeys by two different research groups (2, 3) and was shown to be apathogenic for chickens, and antigenically related to MDV-1 offering good protection against MD (4, 5). For these reasons HVT has been successfully used worldwide as a vaccine against MD in chickens since it was first licensed in 1971 in the United States (reviewed by 3), either alone or in combination with MD vaccines of other serotypes (i.e., attenuated serotype 1 CVI988/Rispens strain or serotype 2 naturally apathogenic strain SB-1) (6, 7). Conventional HVT vaccines (for example, using the FC126 strain) have been used to successfully protect chickens from MD since the early 1970s (8). Several recombinant vaccines using HVT as a vector (rHVT) to express heterologous immunogenic proteins of chicken viruses that cause major diseases such as Newcastle disease (9, 10), infectious bursal disease (11), and infectious laryngotracheitis (12) have been developed since the 1990s, and are used worldwide in the control of MD and of the abovementioned poultry diseases. The Vaxxitek® range of vaccines (Boehringer Ingelheim) and Innovax® range of vaccines (MSD Animal Health) use HVT as a vector to express single genes from other pathogenic avian viruses: infectious bursal disease virus (IBDV), infectious bronchitis virus (IBV), infectious laryngotracheitis virus (ILTV), and Newcastle disease virus (NDV), or combinations thereof. The Vaxxitek® range includes VAXXITEK® HVT + IBD, VAXXITEK® HVT + IBD + ND, and VAXXITEK® HVT + IBD + ILT. The Innovax® range includes Innovax®-ND-ILT, Innovax®-ND-IBD, Innovax®-ND, and Innovax®-ILT.

The efficacy of HVT vaccination against MD has decreased over time, mainly due to increased virulence of MDV-1 strains (13). Nowadays, the use of HVT vaccine alone is restricted to the vaccination of broilers; long-living birds such as breeders and layers are usually vaccinated with a bivalent HVT and CVI988/Rispens vaccine (14). Cell-free lyophilized HVT vaccine, which is cheaper and easier to handle than cell-associated formulations where liquid nitrogen is needed for storage, is frequently adopted for the protection of valuable ornamental chicken flocks with a history of MD, and also some backyard chicken flocks (15).

Both conventional and recombinant HVT vaccines are live vaccines that actively replicate within the host mimicking natural infection and eliciting a protective immune response. HVT vaccinal viruses replicate in the feather follicle epithelium and are persistently shed into the environment through physiological desquamation of epithelial cells (16). Thus, feather tips taken from vaccinated birds represent a non-invasive sample to confirm HVT vaccine administration and uptake for monitoring success of HVT-based MD vaccination in the field (17). Furthermore, the HVT genome can be detected in dust collected from the poultry house environment (18–20), often in combination with MDV-1 and MDV-2 (21).

MD vaccines have been reported as “imperfect” or “leaky”, as they prevent clinical MD but do not impede the infection, replication, and shedding of wild-type MDV-1 into the environment (19, 20, 22–24). Thus, vaccine and field viruses can coexist in the vaccinated host (25) and, in case of mixed infection, molecular tests able to discriminate between MDV-1, MDV-2 and HVT are required.

The full-length genome sequences of the three viral species included in the genus *Mardivirus* are publicly available in online databases (26–31) and many species-specific molecular methods that allow for their differential detection have been developed over time.

Such molecular methods include end-point and real-time PCR assays (17, 18, 25, 32–34) and have one or more of the following drawbacks: they are labor-intensive, require time-consuming post-PCR handling such as gel electrophoresis to visualize the outcome, need expensive specialized equipment, and need to be performed by highly trained personnel. A simple, fast, and accurate test for monitoring of vaccination success in the field could be greatly beneficial for field veterinarians and small laboratories.

Loop-mediated isothermal amplification (LAMP) first described by Notomi et al. (35) and improved by Nagamine et al. (36) is a rapid, extremely specific, and sensitive molecular method that could overcome most of the drawbacks of PCR-based methods. The outstanding specificity is obtained using six primers (two outer primers, two inner primers and two loop primers) that specifically recognize eight different regions in the target genome. A DNA polymerase with strand displacement activity, working under isothermal conditions (temperature between 60 and 65°C) combined with suitably designed primers, enables, starting from the target DNA sequence, the formation of a stem-loop DNA structure, which is the starting point for exponential amplification of the target DNA.

LAMP-based assays for the specific detection of HVT, MDV-1 or MDV-2 genomes have been reported previously (37–43). In all the above-mentioned methods the detection of LAMP products was achieved by sequence-independent methods, such as the utilization of agarose gel electrophoresis or intercalating fluorescent dyes. Sequence-specific detection methods, that enable the exact identification of specific amplicons without being affected by non-specific products (44), are available and, of these, the immunochromatographic lateral flow device (LFD) is one of the most often used. LFDs are designed to specifically detect dual-labeled LAMP DNA amplicons that are captured on a lateral flow test strip, allowing their rapid and direct visualization. Lateral flow tests are low cost, easy to handle, do not require additional equipment, and give an unequivocal positive or negative result that can be interpreted by non-specialist personnel.

The purpose of the current work was to develop HVT-specific LAMP-LFD assays, and to validate these to test field samples in a controlled laboratory setting. Herein, we describe the modification of a previously reported HVT-specific LAMP assay (37), to allow detection of dual-labeled LAMP products with commercially available LFDs. In addition, a novel LAMP assay able to specifically detect the recombinant HVT vaccine VAXXITEK® HVT + IBD was developed and validated. Finally, crude DNA extracted from samples of chicken organs, feathers and poultry house dust subjected to a heat treatment, bypassing the extraction of genomic DNA with commercial extraction kits, was shown to be suitable for virus-specific detection in HVT LAMP-LFD assays.

## Materials and Methods

### DNA Samples From Virus Stocks and Field Samples

All DNA samples tested in this study were already available within the research group. DNA was prepared from chicken embryo fibroblast cells (CEF) infected with *Mardivirus* stocks of known provenance (HVT strain FC126, MDV-2 strain SB-1, very virulent MDV-1 strain RB-1B, and attenuated MDV-1 vaccine strain CVI988/Rispens). Vaxxitek® DNA and Innovax® DNA was prepared from commercial stocks of cell-associated rHVT vaccine VAXXITEK® HVT + IBD (Boehringer Ingelheim), and rHVT vaccine Innovax®-ND-IBD (MSD Animal Health), respectively.

DNA stocks from field samples of chicken feather tips, organs, tumors, and poultry house dust, submitted to the Marek's Disease Virus Reference Laboratory (MDVRL) of The Pirbright Institute between February and December 2020, were also available. The DNA had been extracted from approximately 20 mg tissue or 5 mg dust. These field samples were predominantly from HVT-vaccinated commercial chickens, some of which had been diagnosed with MD, and all samples had already been tested by serotype-specific MDVRL real-time PCR assays to determine C_T_ values for HVT, MDV-2 (25), CVI988/Rispens, and virulent MDV-1 (45). DNA extracted from blood samples of experimental chickens vaccinated with Vaxxitek® or Innovax® was available from a study previously conducted at The Pirbright Institute.

### DNA From Virus BAC Clones

Bacterial-artificial-chromosome (BAC) clones, stable infectious clones of the whole virus genome, generated in the Viral Oncogenesis Group of The Pirbright Institute, were available for HVT FC126 (46) and VAXXITEK® HVT + IBD (unpublished). These BAC stocks were named pHVT-BAC3, and pVaxxitek-BAC, respectively. The number of viral genome copies in BAC DNA can easily be quantified by determining the mean DNA concentration by spectrophotometry and, subsequently, the number of molecules per μl. Therefore, 10-fold serial dilutions of these BAC DNA stocks (10^0^-10^6^ virus genome copies/3 μl), were used to determine the limit of detection (LoD) of each assay.

### Design of LAMP Primers

Primers for the HVT-specific assay were those previously designed and published by Wozniakowski et al. (38) to target eight distinct regions of the HVT070 gene according to the sequence of HVT strain FC126. This gene is unique to HVT and is conserved between all published wild-type HVT sequences available in the GenBank database, and Vaxxitek® and Innovax® [as the HVT070 gene is intact in these recombinant viruses and is not disrupted by insertion of the exogenous gene(s)]. Basic Local Alignment Search Tool (BLAST) search confirmed the specificity of the six primers for the HVT genome.

Primers for the rHVT (VAXXITEK® HVT + IBD)-specific assay were designed using Primer Explorer V5 online software (Eiken Chemical Co. LTD, Tokyo, Japan) with manual adjustments to sequences to improve specificity or sensitivity of the method. The cloning vector sequence and insertion sites of the foreign genes differ between Vaxxitek® and Innovax®, so these vaccine types can be distinguished based on sequence. VAXXITEK® HVT + IBD expresses the VP2 gene of IBDV which is inserted at a specific site in the HVT genome. A new set of LAMP primers was designed to target a distinctive genomic region encompassing the HVT065 gene and intergenic region plus the cloning vector sequence of Vaxxitek®. The sequence required for the primer design was available from previous studies conducted by the Viral Oncogenesis Group of The Pirbright Institute. Each primer was evaluated for GC content, secondary structures, and 3′ or 5′ end stability. Primer specificity was verified *in silico* by BLAST analysis for both the HVT genome and the inserted cloning vector sequence.

LAMP primers, both unlabelled and 5′-labeled with Biotin (5′-Biosg) or 6-Carboxyfluorescein (5′-6-FAM), were manufactured by Integrated DNA Technologies, Inc. (Leuven, Belgium). The sequences for each specific set of primers are given in Table 1. Each primer set consisted of six primers: two outer primers (F3 and B3), two unlabeled or 5′-labeled inner primers (FIP and BIP), and two loop primers (LF and LB).

**Table 1:** LAMP primer sets used in this study.

**Target**	**Primer name**	**Primer sequence and label**	**References**
HVT (HVT070 gene)	HVT-F3	5′-ATAAATTATATCGCTAGGACAGAC-3′	(38)
	HVT-B3	5′-ACGATGTGCTGTCGTCTA-3′	
	HVT-FIP	5′-6-FAM-CCAGGGTATGCATATTCCATAACA GTTTTCCAAACGACCTTTATCCCA-3′	
	HVT-BIP	5′-Biosg-CCAGAAATTGCACGCACGAGTTTT AGAATTTGTGCATTTAGCCTT-3′	
	HVT-LF	5′-TTGAGAAGAGGATCTGACTG-3′	
	HVT-LB	5′-GCGTCATTGGTTTTACATTT-3′	
Vaxxitek® (HVT065 gene and intergenic region + cloning vector)	Vaxxitek-F3	5′-CCGAACAAACTTCATCGCTA-3′	This study
	Vaxxitek-B3	5′-GCTATTGCTTTATTTGTAACCAT-3′	
	Vaxxitek-FIP	5′-6-FAM-CCCAAAGACCTCTATGAACATTTATTTTTGCAAAGAGATGCGTGTG-3′	
	Vaxxitek-BIP	5′-Biosg-TGTCGACTCTAGAGGATCCGAAAATTTTGTTAACAACAACAATTGCATTCA-3′	
	Vaxxitek-LF	5′-TACTCAACGGCGCGTGTA-3′	
	Vaxxitek-LB	5′-CACACCTCCCCCTGAACCTG-3′	

### Real-Time LAMP Assays and Tube LAMP Assays With LFD Readout (LAMP-LFD Assay)

Primer sets were tested in LAMP assays with two different types of result readout, detailed in the following sections. Initially, primers were tested in real-time LAMP as a rapid way to check primer specificity and sensitivity using unlabeled, therefore inexpensive, LAMP primers. Subsequently, 5′-labeled primers were used in LAMP-LFD using in-tube amplification followed by result readout on housed lateral flow test strips.

The real-time LAMP reactions were set up in 96-well PCR plates. Each reaction (total volume of 25 μl) contained the six specific LAMP primers for the virus to be detected (1 μl of 5 μM outer primers, 1 μl of 50 μM inner primers and 1 μl of 25 μM loop primers), 15 μl of GspSSD2.0 Isothermal Mastermix (ISO-004) (OptiGene Limited, Horsham, West Sussex, UK), 4 μl of water and 3 μl of template DNA. An ABI 7500FAST® Real-Time PCR system (Applied Biosystems, Waltham, Massachusetts, USA) was used to amplify and detect the reaction products, under the following thermal cycling conditions: 30 cycles for 1 min at 65°C. The master mix contained a dsDNA-binding dye read by the machine through the SYBR green/FAM detection channel allowing the generation of amplification plots used to identify positive samples. Melt curve analysis was performed at 98°C (15 s), 80°C (1 min), 98°C (1 min), 98°C (30 s) and 80°C (15 s), to confirm reaction specificity for positive samples. ABI 7500 v2.3 software was used to analyse the results.

Once the assays in real-time LAMP were validated, 5′-labeled FIP and BIP primers were ordered for each set of virus-specific LAMP primers. FIP primers were labeled with 6-FAM and BIP primers were labeled with Biosg (Table 1). The “three-stripe LFD strips” used (Abingdon Health, York, UK) have three lines: Test line 1 (T1), Test line 2 (T2), and Run Control line (C). T1 contains antibodies that specifically bind 6-FAM and Biosg to give a chromogenic product to detect 6-FAM/Biosg-labeled amplicons; T2 contains antibodies that specifically bind Digoxigenin (DigN)/Biosg to give a chromogenic product to detect DigN/Biosg-labeled amplicons; C confirms successful running of the reaction solution through the LFD strip. Only two of the three stripes, T1 (marked with a “T” on the plastic housing cassette) and C, were used in our LAMP-LFD assays. LAMP reactions were run in individual tubes. Each reaction (total volume of 25 μl) contained the six specific LAMP primers for the virus to be detected (1 μl of 5 μM outer primers, 1 μl of 50 μM 5′-labeled inner primers and 1 μl of 25 μM loop primers), 15 μl of GspSSD2.0 Isothermal Mastermix (ISO-004), 4 μl of water and 3 μl template DNA. Reactions were run by placing the tubes in a heating block at 65°C for 30 min. LFD strips were assembled into the plastic housing cassettes. Reaction tubes were only opened in a laminar flow cabinet in a designated laboratory, to avoid the risk of laboratory contamination with LAMP amplicons. The whole volume of the LAMP reaction (25 μl) was mixed with 100 μl running buffer and added to the sample application well of the plastic housing cassette. Results were read after 10 min of incubation at ambient temperature.

### Sensitivity and Specificity of the LAMP Assays

The sensitivity of the LAMP assays, tested in triplicate using 10-fold serial dilutions of the BAC DNA stocks, was expressed as limit of detection (LoD) and defined as the lowest amount of analyte in a sample that could be detected by the assay (real-time LAMP or LAMP-LFD) in at least 50% of the replicates. LoD was expressed as absolute copy number of HVT or Vaxxitek® genomes.

To determine the specificity of the assay, DNA from MDV-1 (CVI988/Rispens and RB-1B), MDV-2 (SB-1), HVT (FC126) or rHVT (Vaxxitek® and Innovax®) strains was used as a template for the real-time LAMP and LAMP-LFD assays, and tested in triplicate.

### Crude DNA Preparations

Crude DNA preparations were extracted from ~20 mg samples of chicken organs, feather tips and poultry house dust. The samples were weighed and prepared as 4% (organs and dust) or 8% (feather tips) w/v suspensions in sterile PBS. The suspensions were then vortexed and subjected to a heat treatment in a heating block at 95°C for 10 min, centrifuged at 1,000 × g for 3 min and the supernatant was tested in LAMP-LFD assay as a crude extract.

## Results

### HVT-Specific LAMP Assay: Sensitivity and Specificity

**Figure 1:**
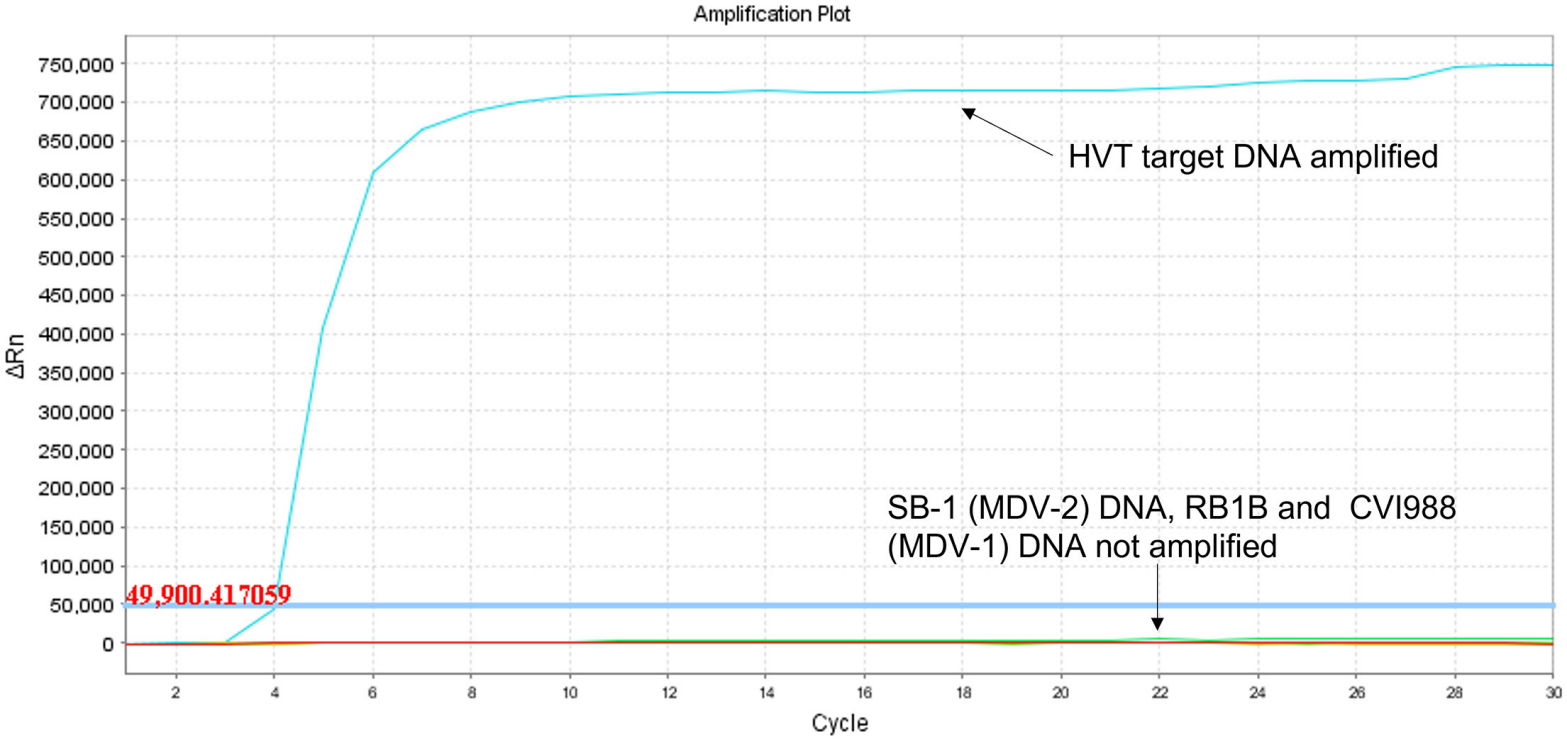
Specificity of HVT LAMP assay in real-time LAMP (amplification plot of fluorescence change vs. cycle number). Specificity of the HVT LAMP primers was tested in real-time LAMP using unlabeled LAMP primers in a 30-cycle assay with SYBR Green readout. *Mardivirus* target DNA was prepared from CEF cells infected with HVT strain FC126, MDV-2 strain SB-1, virulent MDV-1 strain RB-1B, or the MDV-1 vaccine strain CVI988/Rispens. Only HVT DNA was amplified.

**Figure 2:**
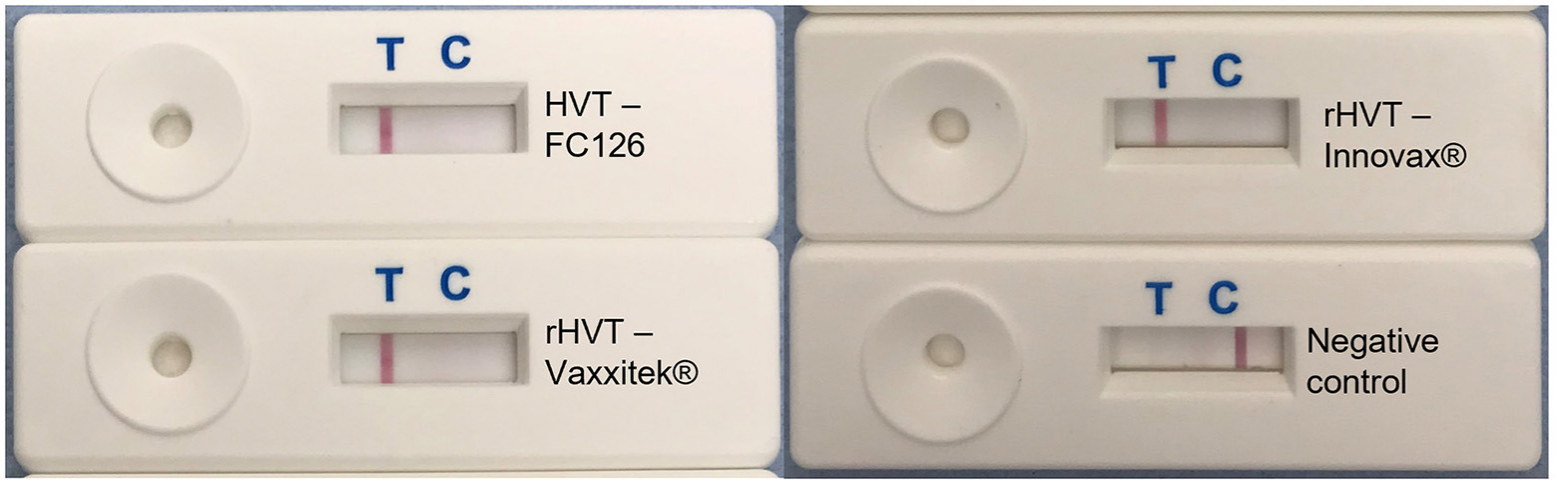
HVT-specific LAMP-LFD assay detects conventional HVT FC126, and the rHVT vaccines Vaxxitek® and Innovax®. The HVT-specific LAMP assay was performed in a heating block and using FAM/Biosg-labeled primers. Target DNA was prepared from non-infected CEF cells (negative control), or CEF cells infected with HVT strain FC126, the Vaxxitek® recombinant HVT vaccine virus, or the Innovax® recombinant HVT vaccine virus. The reaction mixture was loaded onto a LFD for readout of results. A band at the test line (T) shows a positive result, and a band at the control line (C) shows a negative result. HVT FC126, Vaxxitek® and Innovax® all gave a positive result.

Using target DNA from virus-infected CEF cells, the HVT LAMP primer set previously published by Wozniakowski et al. (38), targeting the HVT070 gene of HVT, was confirmed to be specific for the amplification of conventional HVT vaccine (strain FC126) and the recombinant HVT vaccines Vaxxitek® and Innovax® in real-time LAMP (Figure 1) and in LAMP-LFD (Figure 2). Negative results were obtained using DNA from MDV-2 strain SB-1, MDV-1 vaccine strain CVI988/Rispens, and very virulent MDV-1 strain RB-1B.

**Figure 3:**
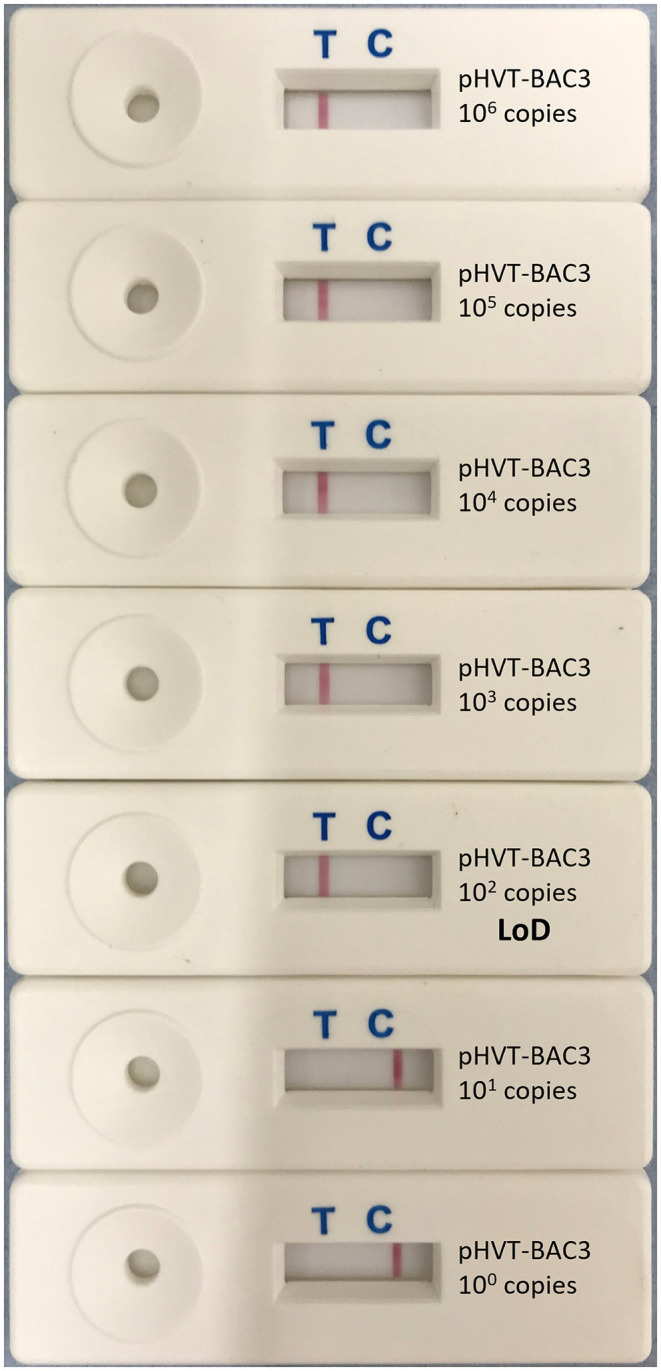
Limit of detection of HVT LAMP-LFD assay. The limit of detection (LoD) was tested using 10-fold serial dilutions of the pHVT-BAC 3 plasmid containing a known number of copies of the HVT genome (10^0^-10^6^). This was repeated in triplicate (replicates not shown). A band at the test line (T) shows a positive result. A band at the control line (C) shows a negative result. The LoD was 10^2^ copies of the HVT genome.

The LoD, determined using 10-fold serial dilutions of pHVT-BAC3 DNA, was 10^2^ copies of the HVT genome in both the real-time LAMP assay and the LAMP-LFD assay (Figure 3). This is 10-fold less sensitive than the HVT-specific real-time PCR assay which targets the HVT sORF1 gene and is an ISO/IEC 17025-accredited assay used by the MDVRL (Table 2).

**Table 2:** Sensitivity of HVT LAMP assays and Vaxxitek®-specific LAMP assays, and comparison with real-time PCR.

**Assay and target gene**	**LoD**^**a**^ **(number of virus genomes)**		
	**Real-time LAMP**	**LAMP-LFD**	**MDVRL real-time PCR**
HVT (HVT070 gene)	10^2^	10^2^	10^1^ (HVT sORF1 gene)
HVT Vaxxitek® (HVT065 gene and intergenic region + cloning vector)	10^2^	10^2^	No Vaxxitek®-specific real-time PCR available

*^a^Limit of detection*.

**Figure 4:**
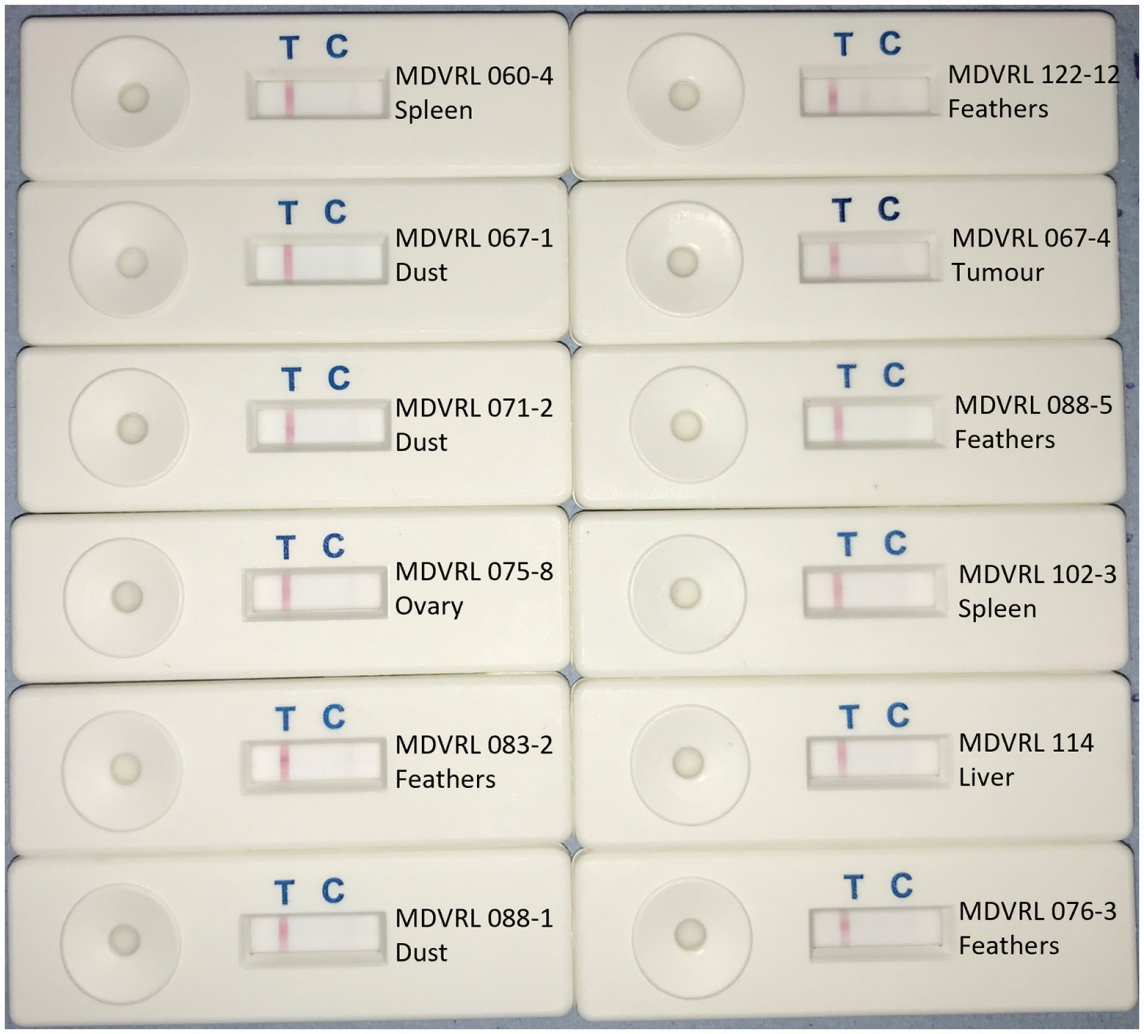
HVT LAMP-LFD assay testing of field samples. DNA was extracted from field samples submitted to the MDV Reference Laboratory for monitoring MD vaccine virus replication and/or presence of virulent MDV field strains. Samples included organs, feather tips, and poultry house dust. A band at the test line (T) shows a positive result. A band at the control line (C) shows a negative result.

The LAMP-LFD results for DNA extracted by commercial kit from 15 field samples are shown in Table 3, and compared with C_T_ values obtained in the MDVRL HVT real-time PCR using the same DNA samples. The sample types included spleens, tumors, feather tips, and poultry house dust. Most samples were from commercial chickens vaccinated with conventional HVT or Innovax®. Thus, all were positive for HVT by real-time PCR, although the C_T_ values varied from 27 to 38. Samples with a C_T_ value <34 were always positive in HVT-specific LAMP-LFD (Figure 4). When the C_T_ value was > 35, some samples tested positive by LAMP-LFD (sample MDVRL067-1) and some negative (samples MDVRL091-1 and MDVRL091-9, result not shown), consistent with the finding that real-time PCR was more sensitive than the LAMP-LFD assay. The latter samples were positive for high levels of MDV-1 and MDV-2 by real-time PCR, so the negative result in HVT LAMP-LFD shows there was no false detection of other *Mardiviruses* in these field samples.

**Table 3:** Field samples tested in HVT LAMP-LFD assay: comparison of LAMP-LFD and real-time PCR results.

**MDVRL sample ref^**a**^**	**Sample type**	**Bird age and type**	**MD vaccination history and clinical signs^**b**^**	**Real-time PCR results^**c**^**	**C_**T**_ in HVT real-time PCR^**d**^**	**HVT LAMP-LFD result**	
						**Purified DNA**	**Crude sample**
MDVRL060-4	Spleen	30 weeks Commercial layer	Vaccinated (no detail) High mortality, splenomegaly	HVT-pos^e^ CVI988-neg vMDV-pos	27.0	Pos	Pos
MDVRL067-1	Poultry dust	33 weeks Commercial layer	Vaccinated with CVI988 + HVT High mortality	HVT-inc CVI988-pos vMDV-neg MDV2-pos	38.6	Pos	Neg
MDVRL067-4	Poultry dust	33 weeks Commercial layer	Vaccinated with CVI988 + HVT High mortality	HVT-pos CVI988-pos vMDV-neg MDV2-pos	33.8	Pos	NT^f^
MDVRL071-2	Poultry dust	20 weeks Breed not recorded	Vaccinated with CVI988 + HVT Outbreak of MD from 18 weeks	HVT-pos CVI988-pos vMDV-pos	30.2	Pos	Pos
MDVRL075-8	Ovary tumor	25 weeks Broiler-breeder	Vaccinated with CVI988 + HVT Visceral tumors from 20 weeks	HVT-pos CVI988-neg vMDV-pos	28.1	Pos	Pos
MDVRL076-3	Feathers	30 weeks Commercial layer	Vaccinated with CVI988 + HVT No clinical signs	HVT-pos CVI988-pos	29.1	Pos	Pos
MDVRL082-3	Feathers	10 weeks Broiler-breeder	Vaccinated with CVI988 + HVT	HVT-pos CVI988-pos vMDV-neg	31.3	Pos	NT
MDVRL083-2	Feathers	30 weeks Broiler-breeder	Vaccinated with Innovax®-ILT + CVI988	HVT-pos CVI988-pos vMDV-neg MDV2-pos	29.1	Pos	Pos
MDVRL088-1	Poultry dust	5 weeks Broiler-breeder	Vaccinated with Innovax®-ILT + CVI988 No clinical signs	HVT-pos CVI988-pos vMDV-neg MDV2-pos	29.9	Pos	NT
MDVRL088-5	Feathers	5 weeks Broiler-breeder	Vaccinated with Innovax®-ILT + CVI988 No clinical signs	HVT-pos CVI988-pos vMDV-neg MDV2-neg	32.2	Pos	Pos
MDVRL091-1	Spleen	29 weeks Broiler-breeder	Vaccinated with CVI988 + HVT Clinical signs of MD	HVT-pos CVI988-neg vMDV-pos MDV2-pos	35.5	Neg	NT
MDVRL091-9	Spleen	29 weeks Broiler-breeder	Vaccinated with CVI988 + HVT Clinical signs of MD	HVT-pos CVI988-neg vMDV-pos MDV2-pos	35.7	Neg	NT
MDVRL102-3	Spleen	60 weeks Commercial layer	Vaccinated with CVI988 + HVT High mortality	HVT-pos CVI988-pos vMDV-neg MDV2-pos	29.9	Pos	NT
MDVRL114	Liver	4 years Pet hen	MD vaccination status unknown Lymphoma	HVT-pos vMDV-neg	34.1	Pos	Neg
MDVRL122-12	Feathers	4 weeks Broiler-breeder	Vaccinated with CVI988 + HVT High mortality at 3–5 days only	HVT-pos CVI988-pos vMDV-neg MDV2-neg	27.0	Pos	NT

*^a^Reference number assigned upon receipt of samples by Marek's Disease Virus Reference Laboratory.*

*^b^Information provided by sender of samples.*

*^c^Any or all of four virus-specific real-time PCR tests performed as requested by sender of sample.*

*^d^Real-time PCR targeting HVT sORF1 gene detects conventional HVT, and the recombinant HVTs Vaxxitek® and Innovax®.*

*^e^pos (positive): C_T_ < 37; neg (negative): C_T_ = 40; inc (inconclusive): C_T_ > 37 and <40.*

*^f^Not tested*.

### Vaxxitek®-Specific LAMP Assay: Sensitivity and Specificity

**Figure 5:**
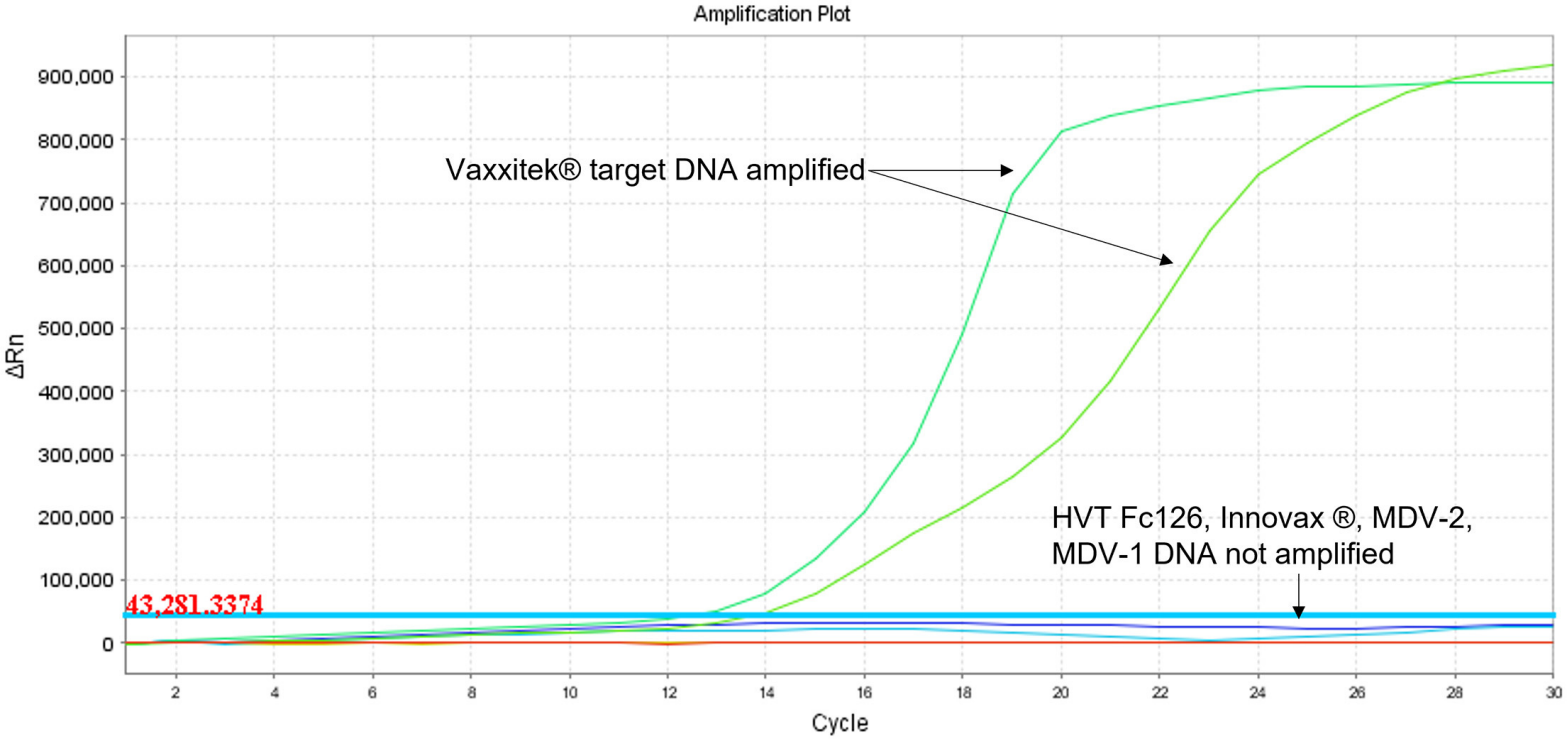
Specificity of Vaxxitek®-specific LAMP assay in real-time LAMP (amplification plot of fluorescence change vs. cycle number). Specificity of the Vaxxitek® LAMP primers was tested in real-time LAMP using unlabeled LAMP primers in a 30-cycle assay with SYBR Green readout. *Mardivirus* target DNA was prepared from CEF cells infected with HVT strain FC126, MDV-2 strain SB-1, virulent MDV-1 strain RB-1B, MDV-1 vaccine strain CVI988/Rispens, Vaxxitek® or Innovax® recombinant HVT vaccine virus. A band at the test line (T) shows a positive result. A band at the control line (C) shows a negative result. Only Vaxxitek® DNA was amplified.

**Figure 6:**
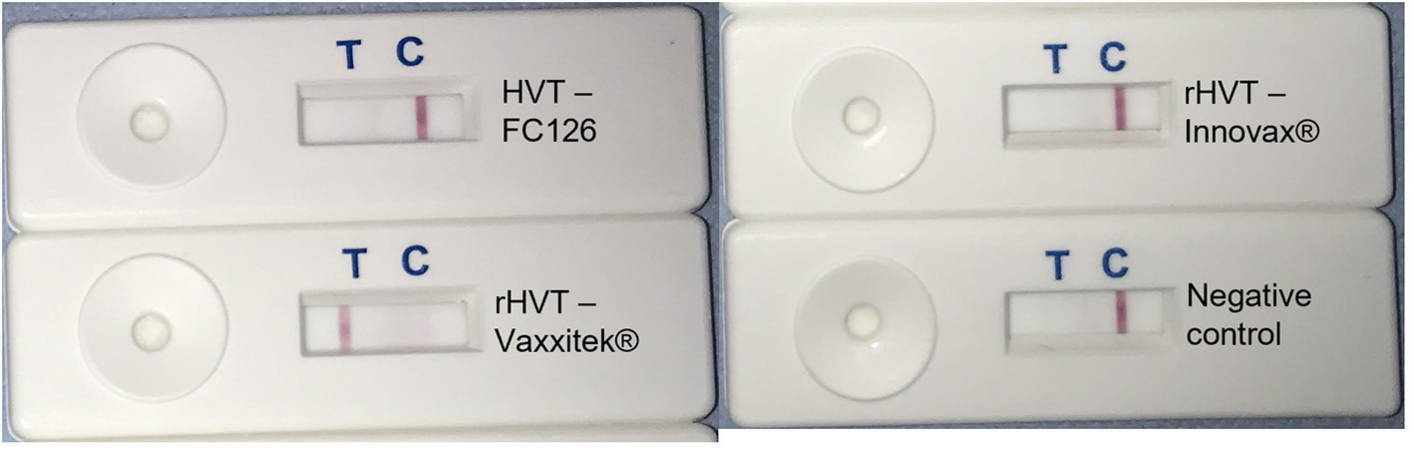
Vaxxitek®-specific LAMP-LFD assay detects Vaxxitek® but not conventional HVT vaccine strain FC126 or rHVT vaccine Innovax®. The Vaxxitek®-specific LAMP assay was performed in a heating block and using FAM/Biosg-labeled primers and target DNA was prepared from non-infected CEF cells (negative control), or CEF cells infected with HVT strain FC126, the Vaxxitek® recombinant HVT vaccine virus, or the Innovax® recombinant HVT vaccine virus. The reaction mixture was loaded onto a LFD for readout of results. A band at the test line (T) shows a positive result. A band at the control line (C) shows a negative result. Only Vaxxitek® gave a positive result.

**Figure 7:**
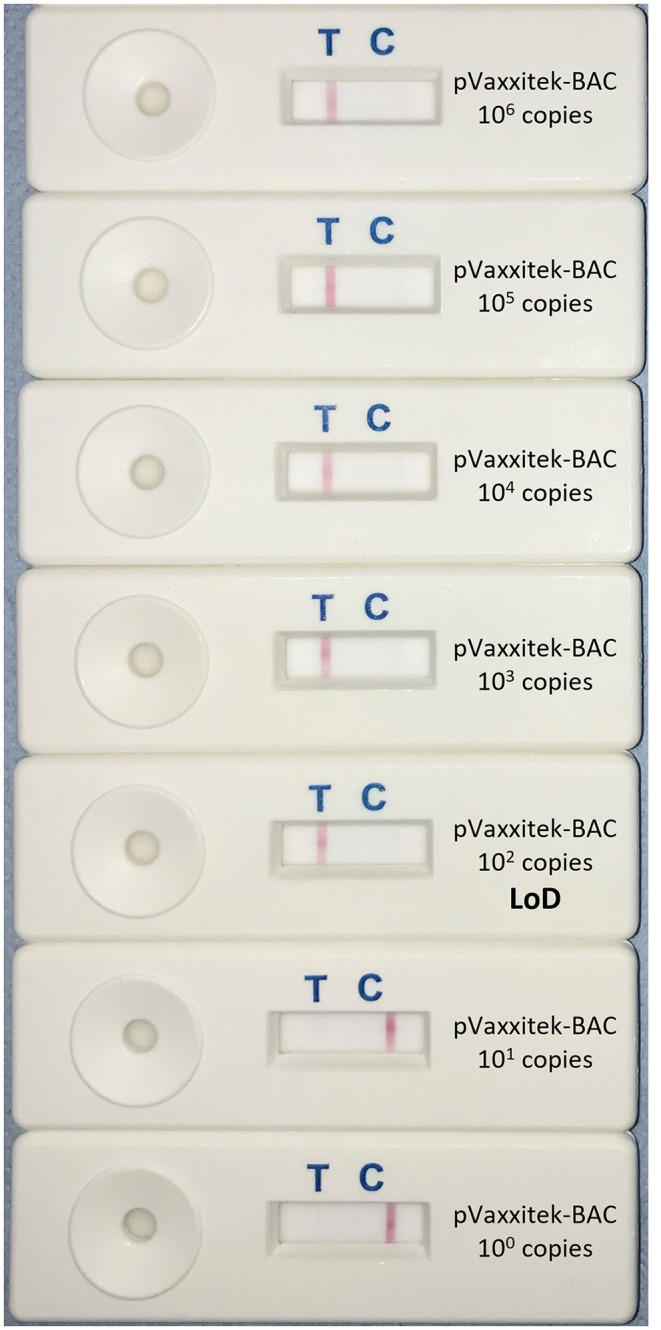
Limit of detection of Vaxxitek®-specific LAMP-LFD assay. The limit of detection (LoD) was tested using 10-fold serial dilutions of the pVaxxitek-BAC plasmid containing a known number of copies of the Vaxxitek® genome (10^0^–10^6^). This was repeated in triplicate (replicates not shown). A band at the test line (T) shows a positive result. A band at the control line (C) shows a negative result. The LoD was 10^2^ copies of the Vaxxitek® genome.

Using target DNA from cell-associated vaccine virus stocks, the assay detected only Vaxxitek® (not conventional HVT vaccine or Innovax®) in real-time LAMP (Figure 5) and LAMP-LFD (Figure 6). The LoD, determined using 10-fold serial dilutions of pVaxxitek-BAC DNA, was 10^2^ copies of the HVT genome in both the real-time LAMP assay and the LAMP-LFD assay (Figure 7). There is no Vaxxitek®-specific real-time PCR assay for comparison; however, the Vaxxitek®-specific LAMP assays were 10-fold less sensitive than the MDVRL HVT-specific real-time PCR assay (Table 2).

No field samples were available from Vaxxitek®-vaccinated flocks. However, DNA samples extracted from blood samples of experimental birds vaccinated with either Vaxxitek® (*n* = 3 birds), or Innovax®, (*n* = 3), or from non-vaccinated control birds (*n* = 3) were tested. The C_T_ values in the MDVRL HVT-specific real-time PCR assay were similar for the three Vaxxitek®-vaccinated chickens and the three Innovax®-vaccinated chickens (C_T_ values all in range 28.7–32.1); however, only the Vaxxitek®-vaccinated samples tested positive by Vaxxitek®-specific LAMP-LFD (data not shown), showing that the Vaxxitek®-specific LAMP-LFD does not detect Innovax® in samples from vaccinated birds.

Furthermore, four field samples from chickens vaccinated with conventional HVT (MDVRL60-4, 82-3, 91-1, and 91-9 (Table 3) were negative by Vaxxitek®-specific LAMP-LFD (data not shown), showing that the Vaxxitek®-specific LAMP-LFD does not detect wild-type HVT in field samples.

### Crude DNA Samples as a Substrate for LAMP-LFD Assay

**Figure 8:**
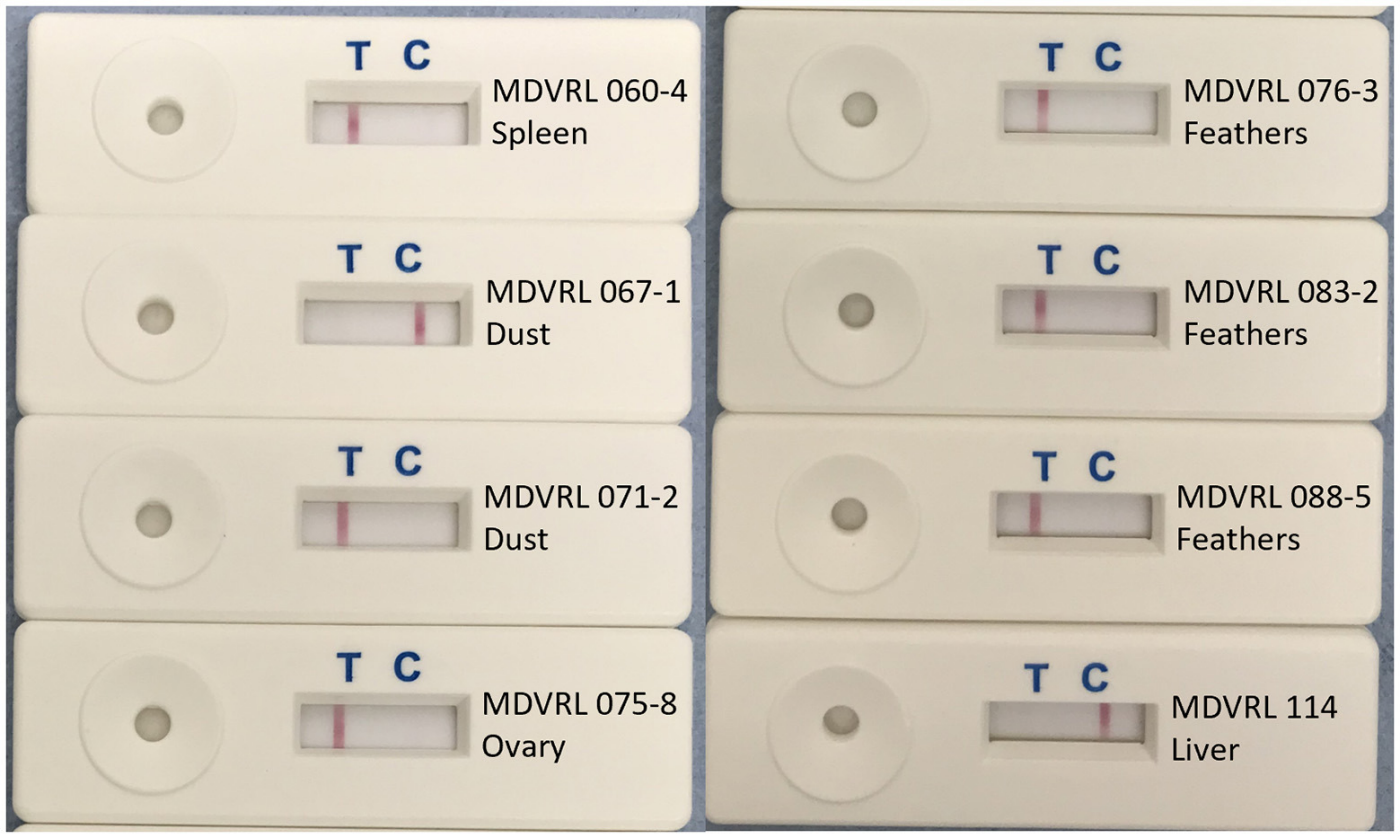
Use of crude DNA to detect HVT in field samples by HVT LAMP-LFD assay. Crude DNA prepared from 20 mg sample material (organs, tumors, feather tips, or poultry house dust) by direct heating was successfully used for detection of HVT by the HVT LAMP-LFD assay. A band at the test line (T) shows a positive result. A band at the control line (C) shows a negative result.

Eight of the 15 field samples, representative of the different sample types (organs, feathers, and poultry house dust) were used to make crude DNA preparations which were then used as template in the HVT-specific LAMP-LFD assay. Six of these eight samples were positive in HVT-specific LAMP-LFD assay when crude DNA preparations were tested, whereas all eight samples were positive when purified DNA was tested (Table 3; Figure 8). The two crude DNA samples which were negative in LAMP-LFD assay were those containing lower levels of HVT (C_T_ > 34 in HVT real-time PCR). Thus, crude DNA preparations from chicken tissues or poultry house dust can be used as a substrate for HVT LAMP-LFD assay, but sensitivity is lower compared with purified DNA substrates extracted from the same original sample.

## Discussion

The present study reports the development of two LAMP-LFD assays for the rapid, sensitive, and species-specific detection of conventional and recombinant HVT-based vaccines, the most commonly used vaccines, worldwide, to prevent and control MD in commercial poultry flocks (47, 48), expanding the diagnostic capabilities, especially in resource-limited settings. The LAMP-LFD technique has proved to be a valuable alternative to the more complex, expensive, and time-consuming PCR-based molecular methods allowing achievement of reliable results in <60 min.

Prior to developing the LAMP-LFD assay, the primer sets were tested in real-time LAMP with fluorescent detection of the LAMP amplicons through the SYBR green fluorescence acquisition channel of the ABI 7500FAST® system. The dsDNA-binding dye included in the master mix intercalates non-specifically into dsDNA, making this method of detection of LAMP products non-sequence specific. For this reason, post-amplification melting-curve analysis was performed to check real-time LAMP reactions for primer-dimer artifacts and to ensure reaction specificity. The perfected real-time LAMP assays were then transposed in LAMP-LFD. Methods for sequence-specific detection, such as LAMP assays coupled with LFD readout, have gained increasing importance in the last few years, because, unlike sequence-independent detection methods used in the previously developed LAMP assays for HVT (37, 43), LAMP-LFD assays are highly specific toward the target DNA (44).

The HVT-specific LAMP-LFD assay was proven to be specific for HVT detection alone and did not cross-react with the other two member species of interest included in the *Mardivirus* genus: MDV-1 and MDV-2. The LAMP primer set used in this assay was previously published by Wozniakowski et al. (38) and further tested by Adedeji et al. (43) which demonstrated that LAMP was a successful alternative to end-point PCR for the detection of HVT in vaccinated and unvaccinated poultry, having much higher sensitivity compared with the end-point PCR assays. This study revealed that the newly developed HVT-specific LAMP-LFD assay was 10-fold less sensitive than the MDVRL real-time PCR assay, which detects the HVT sORF1 gene (25). Despite this, the assay reliably detected HVT in all the tested samples (tissues, feathers, and poultry house dust) from HVT-vaccinated chickens when the C_T_ value (from the ISO/IEC 17025-accredited MDVRL HVT-specific real-time PCR assay) was < 34. Results were variable in samples with a C_T_ > 34.

The results of the Vaxxitek®-specific LAMP-LFD assay confirmed that the assay was specific for VAXXITEK® HVT - IBD detection and did not detect MDV-1, MDV-2, and, more importantly, conventional HVT vaccines or other rHVT vaccines (e.g., Innovax®). The expression cassettes with foreign genes encoding immunogenic viral proteins inserted in the HVT genome and their sequences differ between recombinant vaccines produced by different pharmaceutical companies and are not present in conventional HVT vaccine strains ensuring the differentiation of the different vaccine strains based on their sequence (49, 50). The three-in-one vaccines recently added to the Vaxxitek® range (VAXXITEK® HVT + IBD + ND, and VAXXITEK® HVT + IBD + ILT) use the same bioengineering platform as VAXXITEK® HVT + IBD, so we predict that our Vaxxitek®-specific LAMP-LFD assay will detect all Vaxxitek® vaccines. The LAMP-LFD assay for the detection of Vaxxitek® reliably detected as few as 100 copies of pVaxxitek-BAC DNA per reaction, and gave a positive result only when samples were from birds vaccinated with Vaxxitek®. Unfortunately, no Vaxxitek®-specific real-time PCR assay was available for comparison of analytical sensitivity.

In negative samples, the control line C was very clear on the LFD strips. However, in positive samples with a clear T line, the C line was not visible: the result was either a T line or a C line but not both (an either/or result). This is likely to be because the colored dye/bead mix is the limiting factor in the reaction; when a sample is highly positive, all the beads are captured at the T line and there are no excess beads to migrate to the C line. If a test sample was only weakly positive, so giving a faint T line, there may be sufficient beads to show the C line. Other users of similar LFDs have shown that the intensity of the C line is weak in samples with an intense T line (51).

LAMP-LFD was found to be an effective, sensitive and 100% specific technique for HVT detection even in field samples harboring mixed *Mardivirus* infections, that are very common in the field. In fact, multiple MD vaccines of different serotypes are frequently administered in combination to achieve optimal protection against MD and, furthermore, these imperfect vaccines are unable to prevent superinfections with field MDV strains (19, 20, 22–25). Therefore, the absolute specificity of these HVT LAMP-LFD assays is crucial for their effective application in the field.

It has previously been shown that LAMP amplification tolerates higher levels of inhibitors present in biological samples than PCR (52–54). For poultry samples, these inhibitors include melanin pigment in feathers from colored birds, and particles of dried litter, feces, and feed in poultry house dust. The HVT-specific LAMP-LFD assay developed in this study efficiently amplified and detected DNA from crude organ, feather, and dust samples processed by direct heating, showing robustness to sample-derived inhibitors, but (compared with use of purified DNA) it was slightly less sensitive for detection in samples having low levels of HVT. This treatment of field samples allows further reduction of the overall procedure time by eliminating the need for nucleic acid extraction with commercial kits and demonstrating LAMP-LFD suitability for field use.

In summary, we developed and validated novel HVT-specific LAMP-LFD assays and demonstrated the utility of these assays in a controlled laboratory setting to detect HVT vaccines in field samples. These assays are simple, cost-effective, specific, and sensitive alternatives to PCR-based methods for the rapid and reliable detection of HVT from chicken tissues and feathers and from poultry dust. To our knowledge, this is the first time that LAMP technology coupled with LFD readout has been used for the rapid detection of HVT and Vaxxitek®.

Larger scale testing in the field was not within the scope of this work, but would be required to further validate use of these assays to monitor Marek's disease vaccination directly in the field or in small laboratories with few resources.

Further research will be aimed to develop new LAMP-LFD assays to detect the remaining HVT recombinant vaccines and ultimately to determine the performance of the HVT LAMP-LFD assays in analyzing field samples obtained from poultry flocks vaccinated with different vaccines and vaccination protocols.

## Data Availability Statement

The original contributions presented in the study are included in the article/supplementary material, further inquiries can be directed to the corresponding authors.

## Ethics Statement

Ethical review and approval was not required because no samples were collected specifically for use in this study. The majority of samples tested in this study were from commercial or backyard chickens and were submitted to the Marek's Disease Virus Reference Laboratory at The Pirbright Institute by poultry veterinary surgeons for diagnostic testing. Written informed consent for participation was not obtained from the owners because no samples were collected specifically for use in this study. A condition of sample submission is that the submitter accepts that samples may be retained and used (anonymously) for research purposes. Six samples were from a vaccination experiment previously conducted at The Pirbright Institute.

## Author Contributions

The research project was conceived by VN and SB. The experimental work was conducted by GM (a student from the laboratory of EC) during a 6-month research visit to the Viral Oncogenesis Group at The Pirbright Institute. The manuscript was written by GM and SB, with input from VN and EC. All authors agree to be accountable for the content of the work.

## Funding

This research was funded by a ISCG Agri-food Technology Seeding Catalyst Award (grant reference BB/SCA/Pirbright/17). Research in this paper was also supported by the BBSRC grants BBS/E/I/00007034 and BBS/E/I/00007039.

## Conflict of Interest

The authors declare that the research was conducted in the absence of any commercial or financial relationships that could be construed as a potential conflict of interest.

## Publisher's Note

All claims expressed in this article are solely those of the authors and do not necessarily represent those of their affiliated organizations, or those of the publisher, the editors and the reviewers. Any product that may be evaluated in this article, or claim that may be made by its manufacturer, is not guaranteed or endorsed by the publisher.
